# Important and specific role for basophils in acute allergic reactions

**DOI:** 10.1111/cea.13117

**Published:** 2018-03-23

**Authors:** P. Korošec, B. F. Gibbs, M. Rijavec, A. Custovic, P. J. Turner

**Affiliations:** ^1^ University Clinic of Respiratory and Allergic Diseases Golnik Golnik Slovenia; ^2^ Department of Dermatology and Allergology Carl von Ossietzky University of Oldenburg Oldenburg Germany; ^3^ Section of Paediatrics and MRC and Asthma UK Centre in Allergic Mechanisms of Asthma Imperial College London London UK

## Abstract

IgE‐mediated allergic reactions involve the activation of effector cells, predominantly through the high‐affinity IgE receptor (FcεRI) on mast cells and basophils. Although the mast cell is considered the major effector cell during acute allergic reactions, more recent studies indicate a potentially important and specific role for basophils and their migration which occurs rapidly upon allergen challenge in humans undergoing anaphylaxis. We review the evidence for a role of basophils in contributing to clinical symptoms of anaphylaxis and discuss the possibility that basophil trafficking during anaphylaxis might be a pathogenic (to target organs) or protective (preventing degranulation in circulation) response. Finally, we examine the potential role of basophils in asthma exacerbations. Understanding the factors that regulate basophil trafficking and activation might lead to new diagnostic and therapeutic strategies in anaphylaxis and asthma.

## 1 INTRODUCTION

### 1.1 Of mice and not men: the relevance of murine basophils to human basophils

**HIGHLIGHTS**

Basophils in mice display substantial differences in morphology, function and immunomodulatory roles in comparison with human basophils. This highlights major pitfalls in extrapolating from animal basophil models to acute allergic reactions in humans.^1^, ^2^

Despite an increasing number of studies using mouse models demonstrating an important role for basophils in orchestrating pro‐allergic Th2‐type immune responses and mediating chronic allergic inflammation, extrapolation to humans is highly problematical (Table 1). This is because of substantial differences in basophil morphology and relative expressions of various cell surface receptors, as well as different outcomes of their subsequent stimulation.^1^, ^2^ While recent studies suggest that murine basophils produce similar inflammatory mediators to human basophils,^3^ sensitivities to the biological effects of these mediators differ from one species to another. For example, Berman & Munoz showed that the LD_50_ of histamine (thought to be an important mediator of anaphylaxis) in mice was >20 mg/mouse 4—a sensitivity several orders of magnitude lower than that in humans. This may have contributed to the relative paucity of studies assessing the role of basophils in anaphylaxis, given that basophils are relatively uncommon in comparison with their tissue‐fixed mast cell counterparts in both mice and humans. However, despite their relative rarity, human basophils are at least one order of magnitude more sensitive to IgE‐mediated provocation than mast cells.^5^

**Table 1:** Differences in the pathophysiology of anaphylaxis in murine models compared to humans (adapted from Turner and Campbell^113^)

Murine models	Mediators and mechanisms	Humans
Polymeric IgA (low serum levels) IgD, IgE, IgMIgG1, IgG2a, IgG2b, IgG3	Immunoglobulins	Monomeric IgA, 2 serotypes (IgA_1_, IgA_2_), IgA_1_ abundant in serumIgD, IgE, IgMIgG1, IgG2, IgG3, IgG4
Yes	High‐affinity IgE receptor (FcεRI) on mast cells and basophils	Yes
No	FcεRI receptor on antigen‐presenting cells	Yes
Yes	IgE‐dependent anaphylaxis	Yes
Yes	IgG‐dependent anaphylaxis	No evidence for IgG‐mediated activation of human mast cells. If present, likely to require very high levels of antigen exposure
Very high: in murine models of peanut allergy, dose/weight equivalent to a human eating ≅1000 peanuts!	Allergen dose required through oral exposure to cause anaphylaxis	Very low doses (mgs), for example, for peanut allergy, 10% of individuals react to 1/70 of a peanut
+	Sensitivity to histamine	++++
Yes	Anaphylaxis inhibited by H1‐antihistamines	Little clinical evidence for this. Significant interspecies differences exist in histamine receptor pharmacology.
Yes	Basophils secrete Platelet Activating Factor (PAF)	Data inconsistent

#### 1.1.1 IgE‐ versus IgG‐mediated anaphylaxis

Multiple pathways of anaphylaxis are described in mice. It has been shown that, upon capture of IgG‐allergen complexes, mouse basophils release platelet activating factor (PAF) that increases vascular permeability, leading to anaphylactic shock. In vivo depletion of basophils protects mice from fatal IgG‐mediated anaphylactic shock, but has no effect on IgE‐mediated anaphylaxis. Thus, Tsujimura and Karasuyama 6, 7 postulated that there are two major distinct pathways of anaphylaxis in mice: one is mediated by basophils, allergen‐IgG‐FcγRII‐III receptor interactions and PAF release, whereas the other is mediated by mast cells, allergen‐IgE‐FcεRI receptor interactions and histamine release. Previous murine studies similarly showed that only mast cells contributed to IgE‐mediated anaphylaxis.^8^, ^9^ There are also alternative IgG pathways of murine anaphylaxis, mediated by IgG‐FcγRIII‐macrophages or IgG‐FcγRIV‐neutrophil interactions.^10^, ^11^ In addition, the role of neutrophils was also demonstrated in peanut‐induced anaphylaxis in mice.^12^ More recently, Finkelman et al reviewed the evidence for IgG versus IgE‐mediated anaphylaxis in mice, arguing that dose of allergen is an important factor in determining the precise mechanism of induction.^13^

In sharp contrast, human basophils cannot be activated through IgG receptors, as their function is inhibited by IgG‐mediated triggering *via* FcγRIIb receptors which are the predominant IgG receptor subtype on these cells.^14^, ^15^ Moreover, allergen‐specific IgG antibodies are of questionable pathogenic relevance 16 and are more associated with blocking the effects of allergen‐specific IgE.^17^, ^18^ Furthermore, there is little evidence that human anaphylaxis is in any way mediated by IgG antibodies in relation to either macrophages or neutrophils. Evidence for PAF production by human (as opposed to murine) basophils is also limited and inconsistent.^19^, ^20^

#### 1.1.2 Antigen presentation

Murine basophils appear to be able to present antigens through MHC class II‐dependent interactions.^21^, ^22^, ^23^ However, the role of murine basophils as IL‐4‐releasing antigen‐presenting cells (APC) is limited by the observations that basophils and dendritic cells (DCs) could efficiently co‐operate, where basophils produce IL‐4, whereas DCs present antigens.^24^, ^25^ Eckl‐Dorna et al^26^ and Kitzmuller et al^27^ compared the antigen‐presenting properties of different human cell types including basophils. Human basophils were not able to present allergens to T lymphocytes, whereas a mixture of APCs depleted of basophils did. Furthermore, human basophils lacked the machinery to uptake, process and present allergens, although a small increase of MHC‐II was seen after incubating the basophils with both IFN‐γ and IL‐3. There are some reports that basophils in patients with systemic lupus erythematosus express MHC‐ II,^28^ but these data are not confirmed in other studies.^29^ In addition, human basophils lack protease‐activated receptor expression (PAR), and PAR ligands fail to induce activation of these cells.^30^ In contrast, PAR activators, such as papain, which have been used in many of the mouse models, are able to elicit murine basophil‐mediated Th2 response.^21^

## 2 THE ROLE OF BASOPHILS IN LOCAL ACUTE ALLERGIC REACTIONS

Local allergen challenge induces a prompt migration of basophils to the site of allergic inflammation.

### 2.1 Nose

Basophils have been identified in the nasal washes of patients with allergic rhinitis (AR) and are thought to be an important source of histamine in responses to allergen challenge.^31^, ^32^ Braunstahl et al demonstrated that segmental bronchoprovocation in non‐asthmatic allergic rhinitis patients affects mast cell and basophil numbers in nasal and bronchial mucosa.^33^ The number of basophils increased significantly after challenge, whereas the numbers of mast cells decreased, probably because of the limited immunohistochemical detection (by tryptase and chymase staining) of mast cells after degranulation. At the same time, this study 33 also demonstrated a decrease in the percentage of blood basophils, which might suggest an influx of basophils from the blood into the nasal and bronchial mucosa after the challenge. Interestingly, successful grass pollen immunotherapy is associated with inhibition of seasonal increases in basophils and eosinophils, but not mast cells or neutrophils, within the nasal epithelium of AR patients.^34^

### 2.2 Skin

The skin might be an important route of allergen exposure,^35^ especially in the case of skin barrier disruption,^36^ and significant increases in the numbers of basophils were previously observed 6 hours after intradermal injection of allergen, 37 or, in patch‐test skin sites, for house dust mite allergen.^38^ Furthermore, basophil infiltration into skin lesions seems to be more common than previously thought, indicating that they may play important roles in a variety of inflammatory skin diseases.^39^ Higher number of basophils were detected in inflammatory skin diseases where eosinophils are present,^39^ and those observations are consistent with a recent study which demonstrated a significant correlation between airway basophils and eosinophils in asthma patients.^40^, ^41^

## 3 ASSESSING THE ROLE OF BASOPHILS IN SYSTEMIC ALLERGIC REACTIONS

### 3.1 Human experimental models of acute allergic reactions

**HIGHLIGHTS**

The combination of controlled allergen challenge and emergency department‐based studies may be the optimal model to investigate anaphylaxis in humans.

#### 3.1.1 Controlled allergen challenge studies

Currently, there are two models for studying anaphylaxis in humans: emergency department (ED) studies and controlled challenge models (mostly to food, but also to *Hymenoptera* venom).^42^ Smith et al 43 performed the first prospective human study during sting challenge‐induced systemic allergic reactions, which was followed by a series of similarly designed studies by van der Linden et al in the 1990s.^44^, ^45^, ^46^ However, for safety reasons, in controlled allergen challenge studies, patients with previous anaphylaxis are often excluded from challenge studies due to the potential for life‐threatening reactions.^47^ Furthermore, in the oral food challenge model, the reaction severity at challenge is also limited by the controlled nature of the challenge (allergen exposure is usually terminated at the onset of objective symptoms) and administration of pharmacologic interventions to treat the symptoms. Consequently, two studies which investigated the role of basophils in human anaphylaxis (after insect sting or food challenge), involved only a very limited number of patients who experienced severe reactions after challenge.^48^, ^49^ However, studies in the challenge setting do have the advantage of allowing comparison with prereaction samples, optimal sampling and controlling potentially confounding factors (including acute treatment, where blood samples can often be taken prior to treatment).^50^

Allergen‐induced reactions often manifest themselves as an early asthmatic response, and bronchial allergen challenge may be another model for study of basophils during the acute allergic reaction.^51^ In addition, nasal allergen challenges could also be employed as an experimental set‐up to study the role of basophils in local allergic reactions.^31^, ^52^

#### 3.1.2 Emergency department‐based studies

The ED‐based anaphylaxis study was first described by Lin et al,^53^ and then adapted by others.^50^, ^54^, ^55^, ^56^ Patients with anaphylaxis are studied prospectively at the time of presentation to the ED, with sample collection typically occurring 1 to 2 hours after onset of symptoms, and usually after initial treatment and stabilization.^50^, ^53^, ^55^, ^56^ Patients with the most severe reactions including hypoxaemia or hypotension can be investigated,^50^, ^56^ although this is typically after initial stabilization and treatment (usually with adrenaline). In the case of field‐treatment of anaphylaxis, patients are very often treated with systemic corticosteroids and antihistamines as well.^50^, ^57^, ^58^ Corticosteroids have broad immunological effects, albeit much delayed compared to other anti‐allergic therapies. With respect to basophils, corticosteroids inhibit their pro‐allergic functions,^50^, ^59^, ^60^ and this might be an important confounder.

## 4 THE ROLE OF BASOPHILS IN ANAPHYLAXIS

### 4.1 Basophil activation

**HIGHLIGHTS**

Studies of anaphylaxis investigating human basophil activation in vivo are required.

#### 4.1.1 Secretion of mediators of allergic inflammation

The current evidence for basophil degranulation resulting in anaphylaxis in humans is very limited. However, there are several important indirect observations. Total tryptase (which is produced by mast cells, but not basophils) is within normal limits in up to 30% of patients with anaphylaxis. The proportion of patients with normal tryptase is even higher in the case of food‐induced anaphylaxis (even when blood samples are optimally timed),^61^ or in the case of positive oral food challenge in which symptoms of anaphylaxis are observed.^62^, ^63^ From these data, some authors speculate that, at least in some patients, the anaphylactic episode may primarily involve basophil and not mast cell degranulation.^42^ However, there are several other possible reasons for this discrepancy. For example, in the case of localized (eg in the gut) rather than generalized mast cell degranulation, tryptase may enter the circulation less efficiently. A further level of complexity is added by reports that some mast cells express less tryptase (ie those present in the respiratory epithelium, alveolar wall and small intestinal mucosa) than others (eg in the skin, heart and perivascular tissue), and that in some subjects tryptase may be eliminated very rapidly.^42^

Several studies have assessed the impact of the anti‐IgE monoclonal antibody omalizumab (which prevents IgE from binding to the high‐affinity IgE receptor) on the acute allergic response to nasal allergen or oral food challenge models, which has allowed an evaluation of the relative contribution of basophils and mast cells.^52^, ^64^ Using titrated skin prick testing to assess mast cell responses, and histamine release assays after in vitro allergen stimulation to assess basophil responses, these studies demonstrated that a reduction in symptoms occurred when the basophil—rather than mast cell—response was reduced. These results indirectly suggest a potentially important role for basophils in acute allergic reactions. However, in vitro stimulation cannot directly show that basophils are involved in acute allergic response in different target organs.

#### 4.1.2 Expression of proteins on the plasma membrane

Allergen stimulation of basophils induces the appearance of a number of plasma proteins 65 that can be detected by mAbs and flow cytometry. Increased cell surface CD63 expression is most commonly used to assess the degranulation of basophils,^66^ for which there are several commercial kits (basophil activation tests).^67^ Additional options to assess basophil activation include measurement of CD203c and CD11b, which are located in a rapidly expressed vesicular compartment that is distinct from the histamine‐containing granules, and CD69, which is not related to secretion but is expressed when basophils are exposed to cytokines, such as IL‐3.^65^

Under in vitro basophil stimulation experiments with different types of stimuli (allergens, anti‐IgE, anti‐FcεRI mAbs or fMLP), upregulation of CD63 generally parallels degranulation and histamine release.^66^, ^67^

However, the situation in vivo is not so clear. Turner et al^68^ reported increased expressions of CD63, CD107a and CD203c on basophils following double‐blind, placebo‐controlled peanut challenge in 13 peanut‐allergic subjects (*P* < .01). This is consistent with data from another food challenge study which included 12 subjects with IgE to galactose‐alpha‐1,3‐galactose who experienced a delayed clinical response to mammalian meat.^49^ Two subjects experienced anaphylaxis, and 8 experienced mild reactions. Nine of those subjects (including 2 asymptomatic) showed increased expression of CD63 (median 30% basophils, range 17%‐67%). However, in the same study, 5 of 13 healthy controls, without IgE to galactose‐alpha‐1,3‐galactose, showed comparable increase in CD63 expression (median 34% basophils, range 17%‐46%) after meat challenge, but without any clinical symptoms.

Gober et al^48^ evaluated 35 subjects after the *in vivo* sting challenge, of whom only 1 had a systemic reaction. Despite a significant difference in clinical presentation, the rise in basophil CD63 expression was similar across the group (~2‐ to 3‐fold) and was not related to the severity of the reaction. Interestingly, in the same study, basophils were also examined after in vitro stimulation with insect venom, and the levels of CD63 expression were much greater than after in vivo challenge. A recent ED study showed only a minor increase in CD63 expression on circulating basophils during anaphylaxis, and only one of 31 predominantly venom‐allergic patients had >15% CD63‐activated basophils, despite the fact that the majority experienced a severe anaphylactic reaction with bronchospasm, airway obstruction, hypoxaemia or hypotension, or collapse.^50^

**Figure 1:**
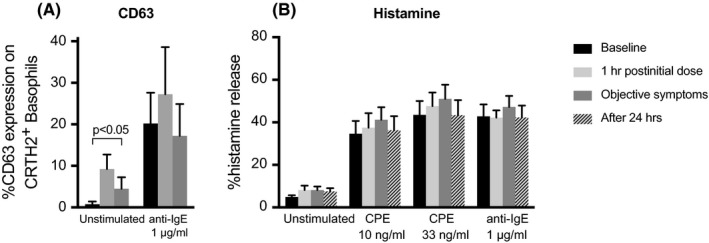
Basophil activation without evidence of degranulation following oral challenge in peanut‐allergic subjects (n = 4). Blood samples were collected prior to, during and 24 h after objective allergic reaction at oral food challenge, as previously described.^50^ Surface expression of CD63 (A) on basophils was evaluated (without further ex vivo stimulation) by flow cytometry.^50^ Basophils were isolated by Ficoll‐density centrifugation and purified to over 90% purity by immunomagnetic cell sorting, using a negative selection technique which we have previously described.^111^ Cells were incubated for 15 min at 37°C before stimulation with crude peanut extract (CPE) or anti‐IgE for 8 min after which histamine release was assessed by spectrofluorometric autoanalysis according to Shore et al 112 (B). Data are shown as mean percentage histamine releases ± SEM. Despite increased surface CD63 expression on ex vivo, unstimulated basophils (A), there was no difference in IgE‐mediated histamine release in the same basophils compared with baseline (B). This implies that circulating basophils have become “activated”—or rather, have increased surface expression of CD63, an activation marker—but with no evidence of degranulation, at least in terms of histamine release. These data presented at the 45th annual meeting of the European Histamine Research Society (EHRS) in Florence, 2016

Vasagar et al^69^ reported that enhanced in vivo surface CD63 expression on circulating basophils was not associated with increased serum histamine levels, although this observation was in patients with chronic idiopathic urticaria rather than an acute allergic reaction. Human studies using in vivo allergen challenge have shown the ability for basophils to demonstrate increased CD63 expression despite the absence of clinical symptoms of an allergic reaction.^48^, ^49^ Moreover, we recently demonstrated a discordance between expression of CD63 on basophils and basophil degranulation (Figure 1).^70^ Thus, the expression of surface activation markers such as CD63 on basophils may not be synonymous with basophil degranulation: the upregulation of activation markers on basophils may be a “bystander” effect—whereby basophils become activated, either due to direct cross‐linking of IgE on the surface, or perhaps due to other mediators (perhaps mast cell‐derived)—without basophil degranulation occurring (at least in terms of histamine release). The basophils might not therefore release inflammatory mediators which themselves contribute to the symptoms of an allergic reaction.

A number of studies have assessed the basophil CD63 response after in vitro stimulation with allergens,^67^ including one which suggested that after the completion of venom immunotherapy, changes in the basophil CD63 expression might reflect the induction of tolerance.^71^ Although these data are interesting, the caveat is that basophil CD63 expression after in vitro stimulation does not readily equate to allergic symptoms and cannot therefore be directly extrapolated to the situation in vivo.

## 5 BASOPHIL MIGRATION

Basophil migration from the circulation might be a key event during anaphylaxis.

### 5.1 The mechanism of transendothelial migration of human basophils

Basophil migration comprises of three sequential steps: adhesion to the vascular endothelium, transendothelial migration and locomotion towards target sites in extravascular tissues. For adhesion to the vascular endothelium, basophils express α_4_β_1_, α_5_β_1_, β_2_ and α_4_β_7_ integrins that interact with different ligands on the endothelium such as VCAM 1, fibronectin and ICAM 1‐3.^72^, ^73^ IL‐3, a major basophil priming and growth factor, can also upregulate the expression of β_2_, and thus augments β_2_ integrin‐mediated adhesiveness for endothelium. Regarding basophil migration and the recruitment of basophils to the sites of allergic inflammation, basophil‐directed chemokines play the most critical roles, by virtue of inducing transendothelial migration and directional movement. It was postulated that CCL2, CCL5 and CCL11 chemokines play a primary role in basophil migration.^72^, ^73^ However, a detailed study by Ikura et al on the migration of freshly isolated human basophils across vascular endothelial cell monolayers showed that the CCR3 ligand CCL11 and the CCR2 ligand CCL2 elicited the most potent migratory response.^74^ Importantly, there was a significant difference in the cellular specificity of these chemokines, as they bind to different chemokine receptors.^73^, ^75^, ^76^, ^77^ CCL11 binds to the chemokine receptor CCR3, which is present on basophils, mast cells and eosinophils. CCL5 binds both to chemokine receptors CCR1 and CCR3, but with higher affinity to CCR1 than to CCR3. CCR1 is also present both on basophils and on eosinophils. CCL2 binds to the chemokine receptor CCR2, which is present on basophils but is undetectable on human eosinophils 78 and it fails to induce eosinophil transendothelial migration. Therefore, in contrast to CCL11 and CCL5, which also induce eosinophil migration, CCL2 preferentially induces basophil migration and may represent a unique mechanism for the selective migration of human basophils.

### 5.2 Migration during anaphylaxis

The results of the experimental allergen challenge in the nose, airways and skin have demonstrated the influx of basophils to inflammatory sites several hours after allergen exposure.^31^, ^37^, ^51^, ^79^ A recent study has suggested that basophils migrate from the circulation during anaphylaxis, both in ED and controlled allergen challenge models.^50^ In the ED study, which included predominantly venom‐allergic adult patients, there was a substantial reduction (80%) in circulating basophils during anaphylactic reactions, and these findings were replicated in peanut‐allergic individuals experiencing allergic reactions during double‐blind placebo‐controlled peanut challenge. In contrast to previous studies which monitored basophils at sites of allergen challenge,^31^, ^37^, ^51^, ^79^ this study^50^ assessed basophils in the peripheral blood, including absolute basophil count measured by flow cytometry using microbeads, with basophils identified as CD123^+^HLA‐DR^‐^ 80 or CRTh2^+^CD303^‐^CD123^+^ cells.^81^ Basophil migration was confirmed using whole blood gene expression analysis of genes which are specific for basophils, including the α‐subunit of the high‐affinity IgE receptor (*FCER1A*)*,* carboxypeptidase A3 (*CPA3*) and histidine decarboxylase (*HDC*).^50^ FcεRI is expressed on mast cells and basophils as tetramers (αβγ_2_) as well as on antigen‐presenting cells, although at substantially lower levels and only as trimmers (αγ_2_).^82^ *CPA3* is expressed in mast cells and basophils and may be expressed in populations of T cell progenitors and thymic T cells.^83^ *HDC* catalyses the formation of histamine from L‐histidine, and in hematopoietic cell lineages, the gene is expressed only in mast cells and basophils.^84^ Importantly, the expression of all three genes significantly decreased during anaphylaxis, and correlated with the absolute number of circulating basophils, indicating that the decrease in whole blood gene expression of *FCER1A*,* CPA3* and *HDC* was due to reduced number of basophils in blood.

### 5.3 The importance of chemokines and allergen‐IgE stimulation for basophil migration

The results of experimental allergen challenge in various organs 31, 37, 51, 79 and recent anaphylaxis studies reveal that basophils migrate during acute allergic reactions. However, the specific mechanism(s) at play causing basophil migration during allergic reactions is unclear. In the previously mentioned study assessing human anaphylaxis,^50^ the major basophil chemotactic factors, including the CCR2 ligand CCL2, and the CCR3 ligands CCL11 and CCL5, were evaluated. Interestingly, during anaphylaxis (in an ED experimental set‐up), only an increase in CCL2 was observed, and increases of this chemokine significantly correlated with a decrease in circulating basophils. The CCL2 increase was also replicated in peanut‐allergic individuals undergoing food challenge. In contrast, no changes were evident for CCL5 and CCL11, which could affect other effector cells such as eosinophils, and no evidence of migration of other cell types (including lymphocytes, neutrophils and eosinophils) was observed. The CCL11 results were consistent with another recent study which demonstrated no changes in CCL11 during anaphylaxis.^55^ These observations suggest that the mechanism of anaphylaxis‐related basophil migration might be CCL2 selective, although the source of CCL2 is currently unknown. It is tempting to speculate that the CCL2 might be mast cell 85 or eosinophils derived.^86^

Suzukawa et al^87^ found that human peripheral basophils migrate in response to IgE‐mediated stimulation, and that the concentrations (of either anti‐IgE, anti‐FcεRI or allergen) required to induce migration are less than that required for degranulation, an observation consistent with previous findings from another research group.^88^ A migration‐enhancing action arising from subthreshold FcεRI cross‐linkage was also demonstrated in murine mast cells.^89^ These observations suggest that IgE‐mediated basophil migration could be induced without activation and degranulation of basophils in circulation. In case of degranulation and histamine release, binding of histamine to H4 receptors further enhances migration.^90^ Repeated exposure over long periods to subthreshold allergen concentrations may also result in basophil desensitization (anergy).^91^, ^92^ The effects of this phenomenon are not yet clear regarding basophil migration. However, we did not observe any basophil desensitization, in terms of histamine release, from the peanut‐allergic donor basophils shown in Figure 1. This may be due phenotypic differences in basophils, the nature of allergen‐IgE interaction and other parameters which still need to be addressed.

### 5.4 What is the clinical relevance of basophil migration?

**Figure 2:**
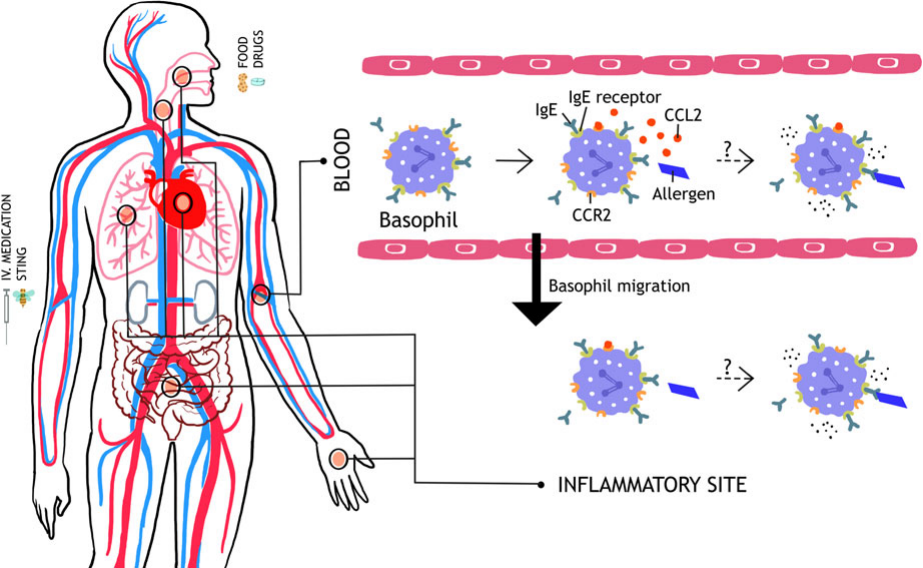
Hypothetical role of basophil migration in anaphylaxis. Upon allergen challenge, basophil‐directed chemokine CCL2 (possibly secreted from mast cells) induces a rapid migration of basophils out of the circulation. This may reactogenic, with migration to target organs resulting in activation and degranulation. Alternatively, the migration may be a protective response, removing basophils from the circulation so that they are unable to degranulate in response to circulating allergen. CCL2, chemokine (C‐C motif) ligand 2; CCR2, C‐C chemokine receptor type 2; IgE, Immunoglobulin E; IgE receptor, high‐affinity IgE receptor (FcεRI)

The importance of basophil migration is currently unclear, and we do not know where or when basophil activation and degranulation occurs during anaphylaxis. The observations that IgE‐mediated basophil migration might occur without degranulation,^87^, ^88^ and that migration out of the circulation may occur at the onset of symptoms,^50^ might be consistent with the hypothesis that basophils migrate to the site of allergen exposure where activation and degranulation could occur, thereby contributing to the clinical presentation (Figure 2). This is consistent with clinical observations of different severities and end‐organ patterns of anaphylaxis which suggest that local rather than generalized mast cell and/or basophil degranulation may predominate in some individuals.^42^

However, it is also possible that basophil migration occurs as a protective response, preventing their activation and degranulation in the circulation which thus limits systemic degranulation and protects patients against severe anaphylaxis (Figure 2). Unfortunately, we are currently unable to answer these essential questions without the labelling of basophils and tracking their migration in vivo during an acute allergic reaction. Such inflammatory cellular migration, in the case of eosinophils and neutrophils, including the kinetics of cellular influx/efflux into the lungs and other organs, was recently studied and imaged over 4 hours *in vivo*, either in control subjects 93 or during allergen challenge in atopic asthmatics.^94^ However, our attempts to undertake similar studies in human volunteers have been limited by poor uptake of radiotracers by human basophils.

## 6 THE ROLE OF BASOPHILS IN ASTHMA

Basophil infiltration in the airways and subsequent activation or immunomodulatory roles might be an important part of asthma pathogenesis and/or exacerbation.

### 6.1 Early and late asthmatic response

Inhalation of allergen leads to an early asthmatic response, which is associated with a decrease in lung function that occurs within 2 hours, caused by the release histamine and cysteinyl leukotrienes from mast cells.^95^ In some patients, the early response is followed by a late asthmatic response, a decline in lung function that occurs during the subsequent 24 hours. The late response is caused by the continued release of mast cell and/or basophil mediators, as well as by the infiltration of inflammatory cells, which produce cytokines and other mediators, resulting in prolonged swelling of the airway mucosa and aggravating of the airway obstruction.^95^

### 6.2 Basophil activation during asthma exacerbations

Previous studies examined the changes in expression of plasma proteins on circulating basophils during asthma exacerbation 96 or after inhalation allergen challenge.^97^ Both scenarios are associated with increased CD203c expression on circulating basophils, but no differences were demonstrated for CD63. Suzuki et al 40 analysed basophils in induced sputum from patients with eosinophilic asthma and showed increased surface expression of both CD203c and CD63. However, this study was performed only on stable patients who had no exacerbations for at least the preceding two months. Salter et al^98^ also demonstrated increased expression of CD203c in blood, bone marrow and sputum basophils after allergen challenge. However, CD203c is located only in a rapidly expressed vesicular compartment that is distinct from the histamine‐containing granules,^65^ and thus, it can be not concluded whether basophil degranulation and/or secretion of immediate mediators occurs during asthma exacerbation.

### 6.3 Migration of basophils during asthma exacerbation

**Figure 3:**
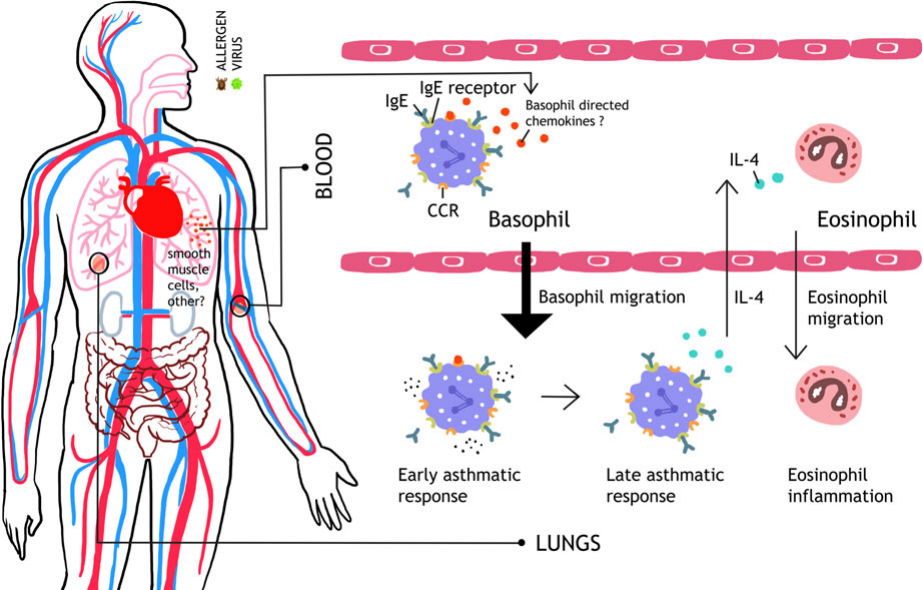
Hypothetical role of basophil migration in asthma exacerbation. Following exposure to allergen or respiratory viral infection, basophil chemotactic factors are released in lungs leading to recruitment of basophils from the circulation to the airways where they may contribute to the early asthmatic response. In some patients, a Th2‐type immune response orchestrated by basophils, mast cells and infiltration of eosinophils can cause late asthmatic response, resulting in prolonged swelling of the airway mucosa and aggravating the airway obstruction. CCL2, chemokine (C‐C motif) ligand 2; CCR2, C‐C chemokine receptor type 2; IgE, Immunoglobulin E; IgE receptor, high‐affinity IgE receptor (FcεRI); IL‐4, interleukin 4

Basophils are increased in induced sputum of asthmatic patients 40, 41, 99 as well as in the sputum or bronchoalveolar lavage (BAL) fluid during exacerbation or after allergen challenge of asthma patients.^51^, ^98^, ^100^, ^101^, ^102^ Basophils were also observed in the lungs of patients with fatal asthma.^103^ This suggests that basophils infiltrate lung tissue in asthma patients. Basophils are increased in the sputum not only from allergic but also of non‐allergic asthmatic patients.^40^, ^41^, ^99^ The highest numbers of basophils were observed in the lungs of patients with eosinophilic asthma, and there is a strong positive correlation between sputum basophil and eosinophil counts.^40^, ^41^ Moreover, Suzuki et al^40^ demonstrated a higher sensitivity, specificity, positive predictive value and negative predictive value of sputum basophil counts for the discrimination of an eosinophilic asthma phenotype than blood eosinophil count and exhaled nitric oxide. As basophil‐derived IL‐4 has been shown to regulate the infiltration of eosinophils,^104^ one could speculate that early basophil migration into the lungs during exacerbations might be important for subsequent infiltration of eosinophils to airway inflammation. Furthermore, basophil‐derived IL‐4 might also play a role for activation of group 2 innate lymphoid cells (ILC2s).^105^, ^106^, ^107^ Hence, human basophils may be essential players in the pathogenesis of asthma (Figure 3).^108^

The source of basophils in the airways of asthmatic patients should be circulating basophils, but there is no current direct experimental evidence which can confirm basophil migration from the circulation to the airways during asthma exacerbations. Assessing the basophil absolute count and/or whole blood expression of genes specific for basophils during asthma exacerbation or after allergen challenge, and comparing them with baseline values, would be an obvious approach. In anaphylaxis models, the induction of migration seems to be related to IgE‐ and FceRI‐cross‐linking upon allergen contact.^87^ However, this might not be the case in asthma, as basophils are also increased in the airways of non‐allergic asthmatics,^40^, ^41^, ^99^ and the most common cause of asthma exacerbations is not allergens but respiratory viral infections. Interestingly, recent reports suggest 109 that for basophil development or homoeostasis, TSLP may play an important role (in addition to IL‐3‐dependent mechanisms), operating in a non‐IgE‐dependent manner. Epithelial cell‐derived TSLP stimulates various aspects of basophil functions including, at least in part, basophil activation in asthma patients, in addition to other important epithelial cytokines (alternatively spliced variants of IL‐33 and IL‐25).^98^, ^99^ Furthermore, it has recently been shown that *in vitro* TSLP‐primed basophil migrate to CCL11 chemokine by upregulation of CCR3 expression.^98^

Finally, basophil chemotactic factors such as CCL2 may also be important for basophil migration in asthma patients, similar to anaphylaxis.^50^ This is supported by recent observations that CCL2 is released by airway smooth muscles in asthma patients, and that levels of CCL2 are increased in the serum of asthma patients.^110^ However, substantially broader studies are required to confirm or refute these speculations.

## 7 CONCLUSIONS

Recent publications have highlighted the importance of human basophils by providing compelling evidence that these cells contribute substantially to anaphylaxis and asthma exacerbations. Understanding the factors that regulate basophil trafficking and activation might lead to new diagnostic and therapeutic strategies in anaphylaxis and asthma.

## ACKNOWLEDGEMENTS

PK and MR are supported by Slovenian Research Agency (reference P3‐0360 and J3‐6787). PJT is in receipt of a Clinician Scientist award funded by the UK Medical Research Council (reference MR/K010468/1). BFG is in receipt of an award funded by the UK Medical Research Council (reference WM/3306381). We are grateful to Dr Mohamed Shamji at Imperial College London for contributing to the data in Figure 1, which was presented at the 45th annual meeting of the European Histamine Research Society (EHRS) in Florence, 2016. PJT and AC are supported by the National Institute for Health Research (NIHR) Biomedical Research Centre based at Imperial College Healthcare NHS Trust and Imperial College London. The views expressed are those of the author(s) and not necessarily those of the NHS, NIHR or the Department of Health.
